# Research on Vegetable Pest Warning System Based on Multidimensional Big Data

**DOI:** 10.3390/insects9020066

**Published:** 2018-06-13

**Authors:** Changzhen Zhang, Jiahao Cai, Deqin Xiao, Yaowen Ye, Mohammad Chehelamirani

**Affiliations:** Mathematics and Informatics College of South China Agricultural University, Guangzhou 510642, China; linzcz333@Gmail.com (C.Z.); stevenfen634606228@Gmail.com (J.C.); dg3195693@Gmail.com (Y.Y.); Chrisamirani@yahoo.com (M.C.)

**Keywords:** pest early warning, data preprocessing, feature selection and extraction, Neural Networks

## Abstract

Pest early warning technology is part of the prerequisite for the timely and effective control of pest outbreaks. Traditional pest warning system with artificial mathematical statistics, radar, and remote sensing has some deficiency in many aspects, such as higher cost, weakness of accuracy, low efficiency, and so on. In this study, Pest image data was collected and information about four major vegetable pests (*Bemisia tabaci* (*Gennadius*), *Phyllotreta striolata* (*Fabricius*), *Plutella xylostella* (*Linnaeus*), and *Frankliniella occidentalis* (*Pergande*) (*Thysanoptera*, *Thripidae*)) in southern China was extracted. A multi-sensor network system was constructed to collect small-scale environmental data on vegetable production sites. The key factors affecting the distribution of pests were discovered by multi-dimensional information, such as soil, environment, eco-climate, and meteorology of vegetable fields, and finally, the vegetable pest warning system that is based on multidimensional big data (*VPWS*-*MBD*) was implemented. Pest and environmental data from Guangzhou Dongsheng Bio-Park were collected from June 2017 to February 2018. The number of pests is classified as level I (0–56), level II (57–131), level III (132–299), and level IV (above 300) by K-Means algorithm. The Pearson correlation coefficient and the grey relational analysis algorithm were used to calculate the five key influence factors of rainfall, soil temperature, air temperature, leaf surface humidity, and soil moisture. Finally, Back Propagation (BP) Neural Network was used for classification prediction. The result shows: I-level warning accuracy was 96.14%, recall rate was 97.56%; II-level pest warning accuracy was 95.34%, the recall rate was 96.45%; III-level pest warning accuracy of 100%, the recall rate was 96.28%; IV-level pest warning accuracy of 100%, recall rate was 100%. It proves that the early warning system can effectively predict vegetable pests and achieve the early warning of vegetable pest’s requirements, with high availability.

## 1. Introduction

China’s vegetable harvesting area is about 24.68 million hectares, with a total output of 758 million tons; the plantation area and total output account for 42% and 65% of the world respectively. China’s vegetable cultivation area, total output, per capita vegetable consumption, and export volume rank first in the world. The damage that is caused by pests and diseases of vegetables is generally up to 20~30%, and in severe situations up to 50% [1]. In southern China, the climate is mild in winter, humid in the summer, and the cultivation of vegetables takes a long time. It is practically feasible throughout the year. At the same time, this climate also caused vegetable pests to occur more frequently and to a greater degree. Especially the major pests of southern vegetables, such as *Bemisia tabaci* (*Gennadius*), *Phyllotreta striolata* (*Fabricius*), *Plutella xylostella* (*Linnaeus*), and *Frankliniella occidentalis* (*Pergande*) (*Thysanoptera, Thripidae*) are highly susceptible to reproduction [2]. The large number of pesticides that are used to control vegetable pests has caused serious vegetable quality and safety problems [3]. The occurrence and development of vegetable pests are affected by many factors, such as the layout of crops, the migration of pests, the diapause patterns, the microclimate of the farmland, and meteorological conditions [4]. The occurrence and succession of pests have become more complicated due to the changes in global climatic conditions and changes in farming systems in different regions. The irrational use of pesticides has severe impacts on natural enemies of pests, destroys the ecological balance, and causes the resurgence of important pests and the outbreak of secondary pests. Therefore, it is hard to explain the outbreak mechanism of pests and to carry out pest warning [5].

The traditional monitoring and early warning technology of vegetable pests is mainly based on the analysis of pest infestation and outbreak mechanism [6], and speculates the comprehensive technology for the distribution and damage trends of pests in a period of time in the future [7]. These need to apply relevant biological, ecological knowledge and mathematical statistics, system analysis, and other methods [8,9]. It usually requires experts to make predictions through biological and mathematical statistical analysis methods [10]. At present, some interdisciplinary and cross-disciplinary technologies, such as insect radar [11], remote sensing monitoring [12,13], and multispectral development [14,15,16] have been developed, which have promoted the rapid development of monitoring and early warning of vegetable pests. However, these methods require strong professional knowledge for analysis and early warning and have low efficiency, large scale, small scope, and poor real-time performance, and it is difficult to meet the requirements of modern agriculture. With the continued maturation of the Internet of Things and big data technology, information can be collected on the integration of various aspects and obtained unexpected conclusions through multidimensional big data analysis [17,18]. Big data has only just started in pest control. There are many problems in the collection, transmission, analysis, and early warning of field data. It still requires a lot of research [19]. The growth, development, reproduction, and distribution of vegetable pests have important relationships with climatic factors and soil environment [20]. Pests need to have a certain range of survival temperature to adapt to and within the appropriate temperature range, the growth rate of pests will accelerate. Therefore, environmental factors have an important relationship with pest occurrence, and it is feasible to use environmental factors for the early warning of pests.

In summary, this study uses small-scale multi-sensor networks to collect substantial data on pests, soil, environment, ecological climate, and meteorology, and conducts research on pest outbreak mechanisms and early warning systems based on multi-dimensional big data. After comparing the classical algorithms used in the previous construction models, we combined the methods of the correlation coefficient, grey relational analysis, and Back Propagation (BP) neural network, a vegetable pest warning system based on multidimensional big data (*VPWS*-*MBD*) was explored. To study the extent of the occurrence of four major pests in southern China vegetables, the framework of the system is described, the process of system construction is explained, and the original algorithm of each sub-module of the system is described. Finally, we have analyzed and evaluated the system performance, summarized the advantages and disadvantages of the system, and proposed further measures for improvement.

## 2. The Architecture of the *VPWS-MBD*

**Figure 1:**
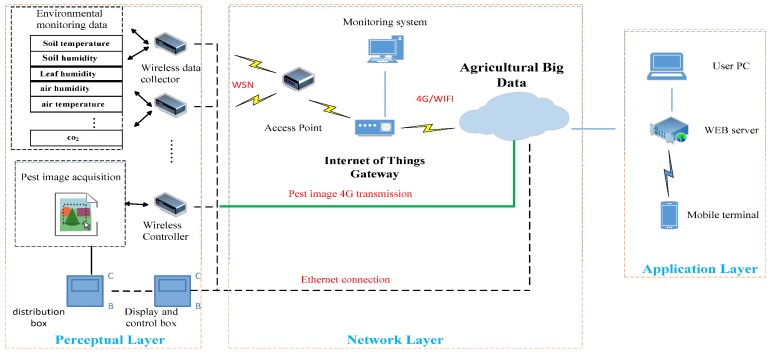
Structure of vegetable pest warning system that is based on multidimensional big data (*VPWS*-*MBD*).

The system contains the collection, transmission, storage, analysis, and distribution of vegetable pest data. As shown in Figure 1, it is divided into a sensory layer, transport layer, and application layer. The sensory layer uses the large-scale data collection of pests, soil, environment, ecological climate, and meteorology at a small scale using a multi-sensor network, which changes the traditional data acquisition model, and greatly increases the amount and accuracy of data acquisition. The transport layer is the data transmission layer. With the help of the 4G network, the collected vegetable pest data is sent to a remote server. At the application layer, users can access the *VPWS*-*MBD* through personal computers, laptops, and smart phones, use farmland sensor control functions and intuitive data visualization capabilities, and conduct pest warnings.

## 3. Detailed Design and Implementation

**Figure 2:**
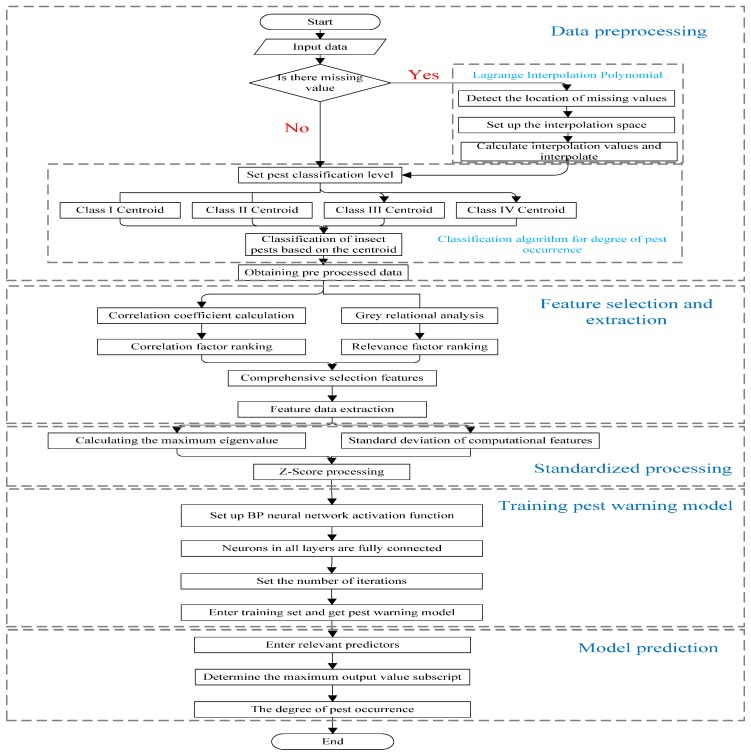
*VPWS*-*MBD* design flow chart.

The design flow chart of *VPWS*-*MBD* is shown in Figure 2. It mainly includes data preprocessing module, feature selection and extraction module, normalization processing module, training pest early warning model module, and model prediction module. Next, the design details of the five core modules of *VPWS*-*MBD* will be introduced in detail. The algorithms involved are all written in the Python programming language.

### 3.1. Data Preprocessing

#### 3.1.1. Discard Missing Values Using Lagrange Interpolation Polynomial

Discard missing values using Lagrange Interpolation Polynomial. In the process of collecting relevant data through multi-dimensional sensors, data loss may occur due to sensor damage and unstable signal transmission. Loss of data is not conducive to the analysis and the training of data. It is necessary to use certain methods to interpolate some missing values. In this study, a missing value processing method based on Lagrange Interpolation Polynomial is used [21].

Scan the original data one by one and see if there are missing values, and label missing and abnormal values. Depending on mathematical knowledge, we can find a *n* − 1 degree polynomial for the known *n* points on the plane.

$$y=a_{0}+a_{1}x+a_{2}x^{2}+\cdots +a_{n-1}x^{n-1}$$

Substituting the coordinates of the *n* points (*x*_1_, *y*_1_), (*x*_2_, *y*_2_), ..., (*x_n_*, *y_n_*) into a polynomial and solving the Lagrange interpolation polynomial is:

$$L(x)\;=\sum_{i=0}^{n}p_{i}\prod_{j=0,j\neq i}^{n}\frac{x-x_{j}}{x_{i}-S_{j}}$$

By substituting the point *x* corresponding to the missing value that has been labeled into the above formula, the approximate value L(x) of the missing value can be obtained.

#### 3.1.2. Classification of Pest Occurrence Based on K-Means Clustering

**Figure 3:**
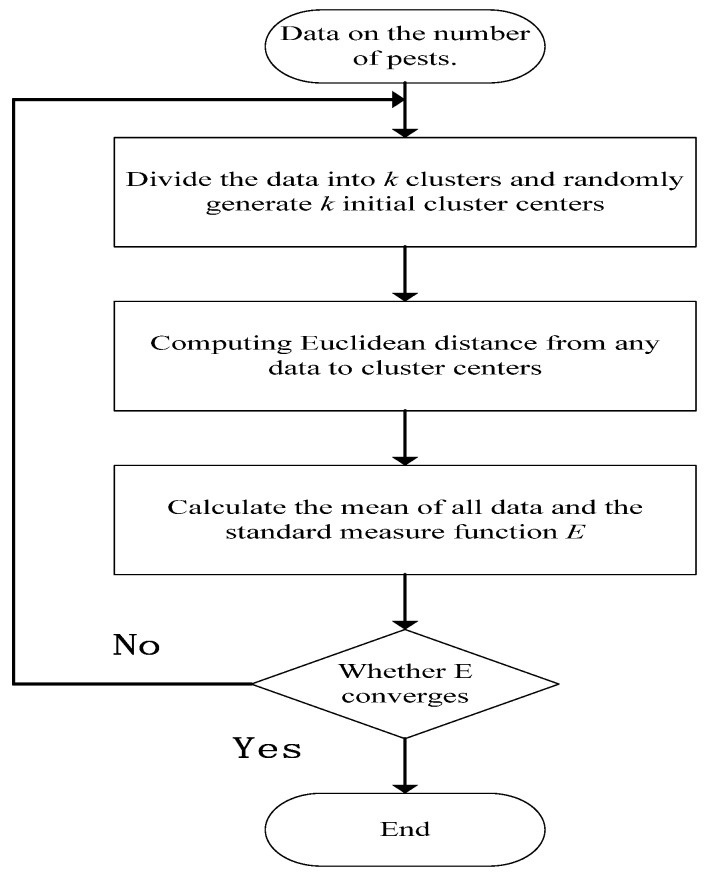
Flow chart of the K-Means clustering algorithm.

The division of the occurrence of pests needs to be divided according to the actual conditions in different places. Different pests are inconsistent in their growth and development in different places. It is very difficult to have a uniform standard. The artificial division of the occurrence of pests can be traced to some extent. There is a rough classification, but the description of the margins between the categories is very vague. Using the K-Means clustering algorithm for discretization [22], the occurrence of pests is divided into multiple levels in the form of maximizing the interval between samples, which avoids subjectivity. The K-Means clustering algorithm flowchart is shown in Figure 3.

The standard measure function is:

$$E=\sum_{i=1}^{k}\sum_{x\in c_{k}}d{\left(d(x,m_{k})\right)}^{2}$$

In the above formula, *c_k_* is the *k*-th cluster, *m_k_* is the centroid of the cluster *c_k_*, *d*(*x*, *m_k_*) is the distance between *x* and the centroid *m_k_*. In the method that is proposed in this study, the Euclidean distance is:

$$d(x,m_{k})=\left\|x,m_{k}\right\|=\sqrt{{\left(x_{1}-m_{k1}\right)}^{2}+{\left(x_{2}-m_{k2}\right)}^{2}+\cdots +{\left(x_{n}-m_{kn}\right)}^{2}}$$

### 3.2. Feature Selection and Extraction

The quality of data and features determines the upper limit of machine learning. Feature selection and extraction can eliminate irrelevant data and redundant data, and can effectively guarantee the efficiency and effectiveness of machine learning. It is an indispensable step in large-scale machine learning. This study selects and extracts the features by correlation coefficient and grey relational analysis.

#### 3.2.1. Pearson Correlation Coefficient

In this study, the Pearson correlation coefficient is first used in the feature selection module in order to calculate the degree of linearity between the data of each dimension and the degree of occurrence of pests, so as to describe the qualitative relationship between the impact factors of pests and the degree of the occurrence of pests. The Pearson correlation coefficient generally analyzes the relationship between two consecutive variables [23], and its calculation formula is as follows:

$$r=\frac{\sum_{i=1}^{n}\left(x_{i}-\bar{x}\right)\left(y_{i}-\bar{y}\right)}{\sqrt{\sum_{i=1}^{n}{\left(x_{i}-\bar{x}\right)}^{2}\sum_{i=1}^{n}{\left(y_{i}-\bar{y}\right)}^{2}}}$$

The range of the correlation coefficient *r* is −1 ≤ *r* ≤ 1. When *|r| >* 0.5, it is a significant linear correlation.

#### 3.2.2. Grey Relational Calculation

The use of correlation coefficient analysis alone has certain flaws. The linear relationship between a single feature and the result can only be qualitatively analyzed from the characteristics and results. Thus, the quantitative analysis between the two is lacking to some extent. Therefore, this study uses the grey correlation analysis method to carry out the quantitative analysis of the characteristics. The grey correlation analysis method determines whether the connection is tight based on the degree of similarity of the sequence curve geometry. The closer the curves, the greater the degree of correlation between the corresponding sequences, and vice versa, the smaller the degree of correlation, based on the degree of similarity or dissimilarity between the trends of factors, as a measure of the degree of correlation between factors [24].

The set of reference sequences is *X*_0_ = {*x*_0_(*k*), *k* = 1, 2, …, *n*}, The sequence of comparison is *X_i_* = {*x*(*k*), *k* = 1, 2, …, *n*}, (*i* = 1, 2, …, *m*). The grey relational degree of *X*_0_ and *X_i_* is defined as:

$$r(x_{0},x_{i})=\frac{1}{n}\sum_{k-1}^{n}r(x_{0}(k),x_{i}(k))$$

In the above formula,

$$r(x_{0}(k),x_{i}(k))=\frac{\underset{i}{\min}\underset{k}{\min}\left|x_{0}(k)-x_{i}(k)\right|+\underset{i}{\partial \max}\underset{k}{\max}\left|x_{0}(k)-x_{i}(k)\right|}{\left|x_{0}(k)-x_{i}(k)\right|+\underset{i}{\partial \max}\underset{k}{\max}\left|x_{0}(k)-x_{i}(k)\right|}$$

In the formula, *∂* is the resolution coefficient and *∂* ∈ (0,1).The grey relational degree *r*(*x*_0_, *x_i_*) of all *m* sequences is ranked from the largest to the smallest, and the correlation order set is obtained. Finally, we can determine the degree of correlation between sequence *X_i_* and *X*_0_.

#### 3.2.3. Key Influence Factor Extraction

After Pearson correlation coefficient and grey relational analysis were calculated for each dimension of vegetable pests’ data, the impact factors of pests and the development trend of pests were described qualitatively and quantitatively. According to the correlation degree, influence factors were extracted, and the specific pest impact factors were selected. The evaluation criteria for key factor is:

$$\begin{array}{c} \begin{array}{cc} E_{k} & = \end{array}\left(\frac{\alpha_{i}-Min\left(\alpha_{i}\right)}{Max\left(\alpha_{i}\right)-Min\left(\alpha_{i}\right)}+\frac{\rho_{i}-Min\left(\rho_{i}\right)}{Max\left(\rho_{i}\right)-Min\left(\rho_{i}\right)},k\right) \end{array}$$

In the formula, $\alpha_{i}$ is the Pearson correlation coefficient sequence and $\rho_{i}$ is the gray correlation factor sequence.

### 3.3. Normalization

Normalization is the basic step of data processing. Because there are different dimensions between different features, the distance between features may vary. If you do not perform the normalization, then it may affect the results of modeling. This study uses a Z-score normalization [25]. After the data is processed, the average value of all feature data is 0, the standard deviation is 1, and its conversion formula is as follows:

$$s_{i}{}^{*}=\frac{s_{i}-\bar{s_{i}}}{\sigma_{i}}$$

In the formula, $\bar{s_{i}}$ is the average value of the feature data and σ is the standard variance of the feature data.

### 3.4. Training Based on BP Neural Network Model

BP Neural Networks have strong nonlinear mapping capabilities and self-learning capabilities. Generalization and fault tolerance are superior to decision trees, logistic regression, and support vector machines, and their effects are relatively accurate As a kind of multi-layer feedforward network trained according to the error back-propagation algorithm, it provides a very robust method for building highly linear partitioned model functions [26].

The BP neural network algorithm is to find the minimum of the error function. Through repeated training of multiple groups of samples, the gradient descent method is used to make the weight change along the negative gradient of the error function, and finally converge to the minimum point [27]. The error function is the following formula:

$$E=\frac{1}{2}\sum_{k=1}^{n}{\left(y_{k}-d_{k}\right)}^{2}$$

In the formula: *n* is the learning sample point, *y_k_* is the actual output, *d_k_* is the ideal output.

The main content of the BP neural network algorithm is to calculate the gradient information of weight between neurons by sample’s learning errors.

$$\delta_{k}=-(y_{k}-d_{k})f^{\prime }({net}_{jk})$$

In the formula: $\delta_{k}$ is the gradient information of the *k* sample learning error function for neuron *k* output and ${net}_{jk}$ represents the input of node *j* when the *k*-th sample is input.

**Figure 4:**
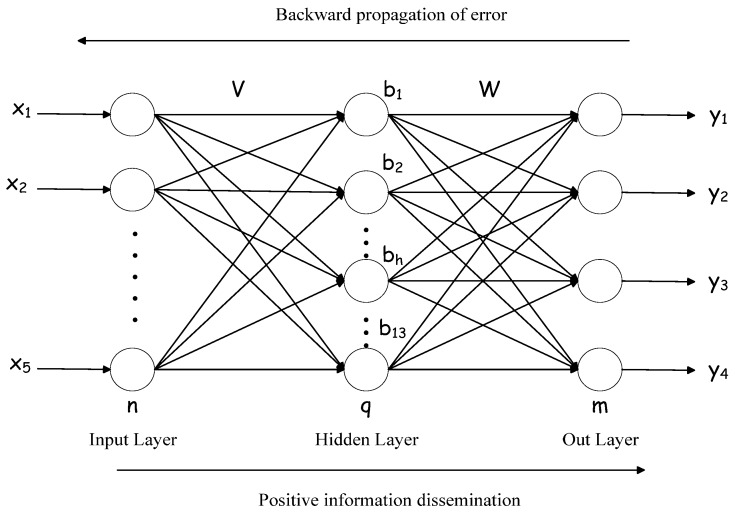
Structure diagram of Back Propagation (BP) neural network.

The occurrence and growth of vegetable pests are complex and affected by multidimensional and complex factors, making BP neural network a great advantage in the classification and warning of vegetable pests. The network model is usually divided into three layers: input layer, hidden layer, and output layer. The network topology is shown in Figure 4.

As shown in Figure 3, *x_i_* is a set of feature vectors, which is obtained from the feature selection and extraction mentioned above, *y_i_* is the level of pest occurrence, obtained by the K-means clustering algorithm in the data preprocessing mentioned above. In the BP neural network model, 134 parameters need to be determined, including 65 weights from the input layer to the hidden layer, 52 weights from the hidden layer to the output layer, 13 hidden layer thresholds, and four output layer thresholds.

Before the BP neural network algorithm starts running, the initial learning times are set to 0 and the upper limit of the learning times is set, and the thresholds and weights are randomly set. Generally, the decimal within the closed interval between −1 and 1 is taken. The input layer accepts the feature set data and performs forward propagation to obtain the parameters of the output layer and the hidden layer. Compare the output layer data calculated by forwarding propagation with the data of the real output layer to obtain the error value between them. The error function is used to determine whether the error is less than the upper limit of error. If the error determined is less than the upper limit of error, then the weight and the threshold of each neuron can be updated by reverse propagation. After updating the thresholds and weights, repeat the above steps again and check whether the number of learning reaches the preset upper limit. If the number of times of learning has reached the preset upper limit, learning stops. When the number of learning has reached the preset upper limit value, the model training is completed.

## 4. Experimental Testing and Analysis

### 4.1. Experimental Data Acquisition Environment Deployment

The test base of this study is in the vegetable garden of Nanshan Base of Dongsheng Farm Co., Ltd. in Guangzhou. The park is managed by professionals and the cultivation is scientific and reasonable. We choose the organic planting of vegetables, not spraying pesticides, as far as possible to ensure the planting environment close to the natural planting environment and reduce the occurrence of pests by human factors. This study uses a data acquisition system that was manufactured by CAIPOS, Inc. in Graz, Austria, which integrates a soil moisture sensor, a soil temperature, an air temperature and humidity sensor, a leaf wetness sensor, and a rain gauge. Specific manufacturer information of all the sensors refers to Table 1.

**Figure 5:**
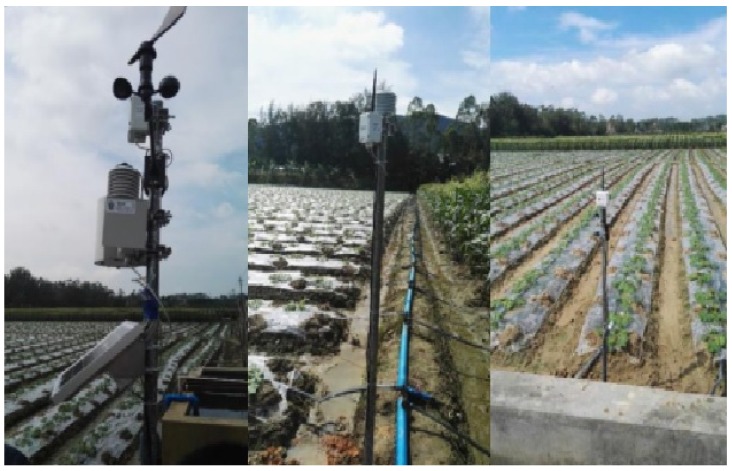
Sensor devices of Caipos.

Two solar stations were installed in the vegetable garden to provide wireless sensors with AC power. The sensors were installed around two solar panels. The signal collection station was installed above the vegetable garden to avoid obstacles. The signal collection station acquires sensor information through both wired and wireless methods, and it sends the information to the server. Field trials are shown in Figure 5.

**Figure 6:**
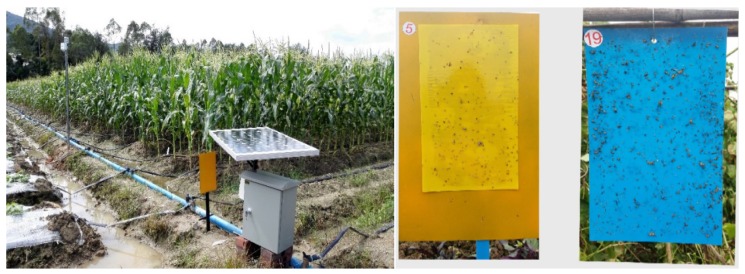
Pests image acquisition equipment.

In order to automatically collect data on vegetable pests in agricultural land for a long period of time, pest trapping and monitoring equipment for vegetables were designed in this experiment. As shown in Figure 6, *Bemisia tabaci* (*Gennadius*), *Phyllotreta striolata* (*Fabricius*), *Plutella xylostella* (*Linnaeus*), and *Frankliniella occidentalis* (*Pergande*) (*Thysanoptera*, *Thripidae*) were collected through yellow traps. We waited for several types of major vegetable pests to take photos of these yellow sticky boards and use the number of pests on the day minus the number of pests on the previous day to calculate the number of new pests on the day. The device includes a solar power supply device, a trapping device, and a monitoring device, and it develops a control program. After the program is started, the program automatically captures the image of the pest according to preset parameters and sends it to the remote server via the 4G network. At the same time, the remote command is obtained, and executes it.

From June 2017 to February 2018, a total of 2536 environmental and pest data were collected, of which 136 were processed for missing values. The collected environmental data combined with the image data of the pest can be obtained, as shown in Table 2.

### 4.2. Evaluation of Pest Levels Based on K-Means Clustering

**Figure 7:**
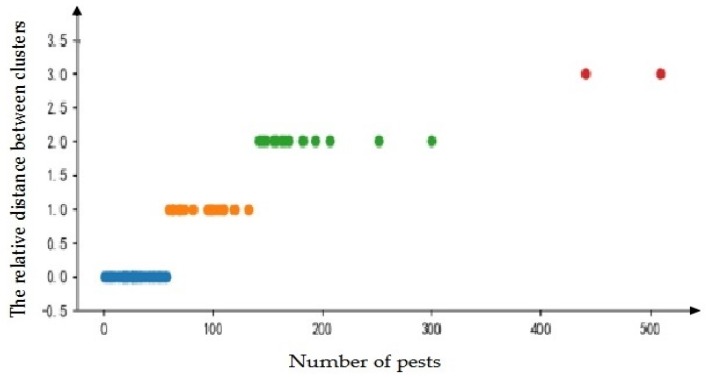
The Division of Pests in Vegetables in South China.

In this experiment, in order to more objectively obtain the level of vegetable pest warning, K-means clustering was used to cluster the number of pests that were collected. The calculated results are shown in Figure 7.

As can be seen from Figure 6, the degree of occurrence of pests can be clearly divided into four levels. The number of pests is classified as level I in 0–56, the number of pests is classified as level II in 57–131, and the number of pests is classified as level III in 132–299. Grades and pests are divided into level IV above 300.

### 4.3. The Effective Calibration of Multi-Dimensional Key Factors

The results that were obtained by calculating the impact factors of pests collected by the Pearson correlation coefficient and Grey relational analysis are shown in Table 3.

Using the southern China vegetable pest optimization feature selection algorithm to evaluate each influencing factor, the results are shown in Table 4.

It is important to select the influencing factors with high correlation to train the prediction model. From Table 3, the impact factor of air humidity is only 0.08. If the feature with too low impact factors is used, prediction model training will be inaccurate. As we expected, the impact factors of leaf surface humidity, soil temperature, air temperature, and rainfall were between 1.11–1.94, which was highly related to pest occurrence. The impact factor of soil moisture was 0.72, which was a little low, but it also meets the model training requirements. Therefore, the feature set of the pest prediction model finally selected the leaf surface humidity, soil temperature, air temperature, rainfall, and soil moisture.

### 4.4. BP Neural Network Training

It was observed that the number of neuron hidden layers is related to the number of input neurons and the number of output neurons. The number of hidden layers is usually set as the sum of the number of input layers and output layers plus 0 to 10 neurons. According to this experience, this study will set the number of neurons in the hidden layer to 13, put the data selected by the features into the BP neural network model, iterative learning 10,000 times, and finally, get the vegetable pest warning model.

### 4.5. Verification of Early Warning Effect of VPWS-MBD

**Figure 8:**
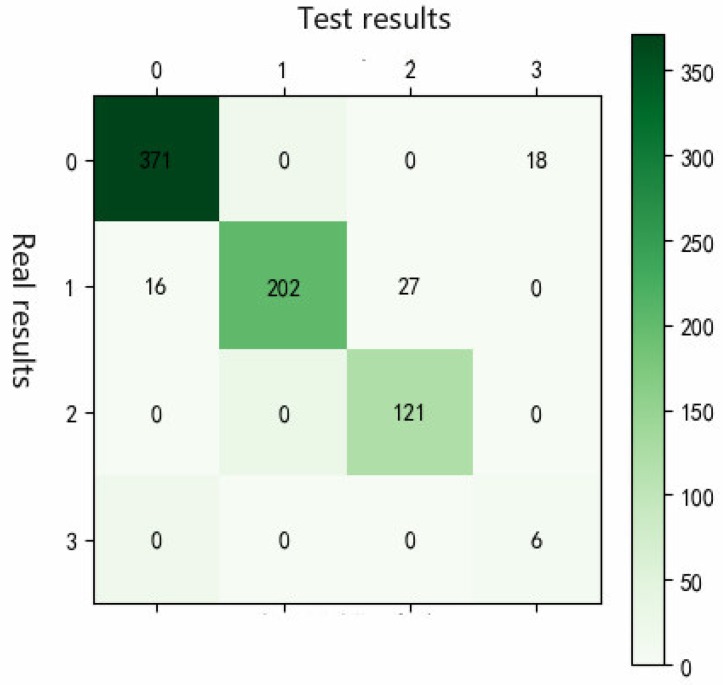
Confusion matrix diagram of warning results.

In this study, 30% of the multidimensional dataset is used for model verification, and the rainfall, soil temperature, air temperature, carbon dioxide concentration, and leaf wetness are selected as the input tuples. The obtained results can be represented by a confusion matrix, as shown in Figure 8.

Each column of the confusion matrix represents a vegetable pest prediction category, the total number of each column indicates the number of data to be predicted to be the category, each row represents the true attribution category of the data, and the total number of data in each row indicates the number of data instances in the category. According to the confusion matrix diagram, the predicted results are compared with the actual results, and the results obtained are shown in Table 5.

It can be seen from Table 4 that the accuracy of early warning models for pests at level I, level II, level III, and level IV is 96%, 95%, 100%, and 100%, respectively, indicating that different pest occurrences are predicted. The accuracy of the early warning is high. At the same time, from the F1 measurement point of view, the early warning model of pests in south China has a good evaluation of the forecasting and forecasting of the degree of occurrence of pests, especially for severe pests, the early warning model will basically not be wrong. The main errors of the analysis and early warning model come from two aspects. The first aspect is that the algorithm has a certain difference between the missing value of the interpolation value and the actual value; in the second aspect, the number of pests is identified by artificial eyes, and there is a certain amount of artificial error.

## 5. Conclusions

Aiming at the disadvantages of large-scale, low-precision, and time-ineffectiveness in vegetable pest warning, a vegetable pest warning system that is based on multi-dimensional big data was designed. A large-scale multi-sensor network was used to collect large data on pests, soil, environment, ecological climate, and weather, and the images of vegetable pests were automatically collected and classified and counted for vegetable pests. A series of algorithms, such as BP neural network machine learning, were used. The training and learning of the model eventually achieved *VPWS*-*MBD*. The experimental results show that *VPWS*-*MBD* has high accuracy and stability, which is conducive to the establishment of scientific and effective prevention and control plans for pests and diseases, scientific guidance of agricultural production, and the improvement of the quality and yield of vegetable pests. All of the data used in this study comes from the real environment of the southern China farmland. The actual environmental data ensures that the prediction results are closer to the actual situation on the ground. At the same time, the characteristics of training can be adjusted according to different data, making the system’s architecture more flexible. The BP neural network has a strong fault tolerance capability. Under the condition of sufficient data, it can obtain excellent prediction results. The system can be further expanded and it can be used to predict and warn any particular pest in the future.

## Figures and Tables

**Table 1:** Caipos Sensors.

	Model	Measurement Range	Accuracy
Soil Moisture	C200A	0% VWC to saturation	2–5% VWC
Soil Temperature	ST	−0 °C + 70 °C	±1 °C%
Air Temperature & Relative Humidity	CaipoRHT	Rel.Hum.: 0.100% Temp.: −5 °C + 60 °C	Rel.Hum.: ±2.5%Temp.: <±0.4 °C
Leaf Wetness	CaipoLWS	0.100%	3%
Rain Gauge	Caipos Rain Gauge	0.2mm	±1%

**Table 2:** Pest monitoring data.

Date	Precipitation	Soil Temperature [°C]	Air Temperature [°C]	Soil Moisture [%]	Leaf Wetness [%]	Air Humidity [%]	Pest Level
2017/11/2	0	24.45	21.71	143.88	6.52	58	1
2017/11/3	0	24.32	20.58	279.29	9.67	66	3
2017/11/4	0	24.36	21.9	42	10.1	78	2
2017/11/5	0	24.64	23.33	143.52	8.04	81	2
2017/11/6	0	24.8	24.04	193.21	11.21	79	0
2017/11/7	0	24.97	22.95	250.52	9.29	81	1
2017/11/8	0	23.98	18	20.62	3.6	76	2
2017/11/9	0.5	25.08	25.41	41.54	11.58	89	2

**Table 3:** Pearson Correlation Coefficient and Grey relational analysis for Southern China Vegetable Pest Data Set Computation Results.

Characteristic Factors	Correlation Coefficient	Correlation
Rainfall	0.283327	0.7125
Air humidity	0.161855	0.621
Air temperature	0.404526	0.7011
Soil temperature	0.465947	0.6903
Soil moisture	0.392791	0.6218
leaf wetness	0.473599	0.75399

**Table 4:** Evaluation Results of Affecting Factors on Vegetable Pests in the South.

Influencing Factors	Impact Factor
Rainfall	1.11
Air humidity	0.08
Soil temperature	1.54
Air temperature	1.37
Soil moisture	0.72
leaf wetness	1.94

**Table 5:** Analysis of Early Warning Results of *VPWS*-*MBD*.

	I	II	III	IV
True positive (TP)	367	206	143	20
False Negative (FN)	11	8	6	0
False positive (FP)	14	11	0	0
True Nagative (TN)	369	536	612	741
Precision (p)	0.96	0.95	1	1
Recall (r)	0.97	0.96	0.96	1
F1 value	0.96	0.95	0.98	1
